# Earache: Its Significance and Early Management

**Published:** 1897-06

**Authors:** Frank Whitehill Hinkel

**Affiliations:** Buffalo, N. Y.; Clinical Professor of Laryngology, University of Buffalo; 305 Delaware Avenue


### Selections and Abstracts.

### EARACHE: ITS SIGNIFICANCE AND EARLY MANAGEMENT.

By FRANK WHITEHILL HINKEL, M. D., Buffalo, N. Y.

Clinical Professor of Laryngology, University of Buffalo.

Pain is so early prominent and constant a symptom in acute catarrhal inflammation of the middle ear that it is regarded by laymen as the disease itself. Common usage of the term "earache" bears witness to this:

It is the purpose of this paper to recall anew to your attention that "earache" has grave significance, involving possible lifelong disability of one of our most important senses, and more frequently than is appreciated, issuing lesions that menace life itself. Especially would I impress upon you that, except ear disease occur as a complication, these serious sequences can be prevented almost invariably if proper treatment be rigidly and accurately installed in the very first stage of the attack. It is important that the family physician—the general practitioner—have clear and positive ideas of the conditions involved and the responsibility that devolves upon him. For these cases are seen, while abortion is yet possible, far oftener by him than by the expert, whose services are usually sought when the disease is fully established. This particularly applies to practice in the country, where early consultation is difficult, often impossible. And, unfortunately, the management of these cases by the family physician often lacks in decision and accuracy. Uncleanly and useless local applications are employed, the real indications are unfulfilled, and the golden opportunity for abortive treatment is lost. For this reason, this simple though important theme is presented before you, and with the greater interest because the proper early management of earache does not involve practised technique nor elaborate apparatus.

By far the greater number of cases of earache occur in childhood, when fortunately the recuperative powers are greatest. If proper and sufficiently early treatment be given these cases, whether of the occasional or recurring type, little or no permanent disability will follow. How weighty a responsibility lies on the attendant in these cases, and how little tendency there is to entire restoration of function in cases entrusted solely or mainly to their natural course, are shown by Hartman's estimate that one in four adults has impairment of

1. Read before the Medical Society of the County of Erie, January 12th, 1897.

hearing in one or both ears. Certainly your observations, like my own, show the great prevalence of defective hearing; and when we remember that considerable deafness may exist in one ear unknown to the individual, if the other ear have normal acuteness, it is easy to appreciate how many cases escape attention. Again, the mortality from cerebral disease, secondary to acute or chronic purulent otitis, is not inconsiderable. It has been estimated at 3-10 of 1 per mille of all deaths. Some insurance companies regard a chronic purulent otitis as a cause for rejection as a bad risk.

In view of the frequency and gravity of these sequences of neglected inflammations of the middle ear, and remembering the almost unvarying recovery of function (in acute cases not complicating the fevers) under correct treatment—if that treatment be given *early* in the attack—no further argument can be necessary to urge the importance of rendering this aid promptly and efficiently.

In earaches that occur but once in the individual the significance as to etiology is neither constant nor often of much importance. It is otherwise, however, where the attacks recur with each winter's stormy weather, or with every "cold." Here the predisposing cause must be removed, if treatment is to avail to preserve the hearing. The offending lesion in these cases will be found to be almost invariably a hypertrophy of the lymphoid tissues that encircle the upper air and food tracts. So constant is the relationship between "adenoids" and recurring earaches in children that the discovery of the one should always lead to investigation of the other. Earaches in childhood should always suggest a careful examination of the naso-pharynx. It is surprising in how young a child a visual examination of the vault of the pharynx can be made with the mirror. When that fails, for any reason, a digital exploration can always be made. If the examining finger have a practiced touch, much can be learned in this way. Remember that the faucial tonsils may be entirely normal in size and appearance, while the pharyngeal tonsil may be considerably diseased and hypertrophied. Remember, also, that this lymphoid hypertrophy in the naso-pharynx may be too slight to cause the typical symptoms or "adenoids," such as mouth-breathing, and the like, and yet by its location induce repeated inflammations of the middle ear.

Hypertrophy and inflammation of the tonsils, both faucial and pharyngeal, act deleteriously upon the ear in two ways—first, by extension from them to the middle ear through the Eustachian tube of congestion or of infectious inflammation; and, second, by mechanical interference with the action of the soft palate. The diaphragm-like sheet of muscular tissue, that we call the soft palate, is inserted into the membranous and cartilaginous portions of the Eustachian tube, as well as to the petrous portion of the temporal bone. Like other structures of the upper air and food tract, it has varying functions and performs a part in several special mechanisms. It is thus a part of the mechanism of the middle ear, and is the means of ventilating and equalizing air pressure in that important chamber. Every time we swallow or speak, the contractions of the soft palate draw upon parts of the tubes and open their usually closed lumen to the passage in or out of air. If the palatal action be hampered by swollen tonsils, or adenoid hypertrophies, or if these adenoids themselves so press upon the Eustachian orifices as to prevent their dilating, the ear function is interfered with and a state of deranged tension of the ossicles and drum membrane is induced, with more or less passive congestion of the mucous membrane of the tympanum. Acute inflammations readily occur, causing the familiar earaches. The inflammation never entirely subsides and the hearing sooner or later suffers. It is plain that no treatment will be successful that does not restore freedom of action to the soft palate and diminish the extent and frequency of inflammatory attacks in the naso-pharynx. I will not enlarge upon the treatment of lymphoid hypertrophy of the pharynx. Thorough removal of the hypertrophied tissue alone can be relied upon to restore the much-needed function of the soft palate and to relieve the engorgement of the middle ear. If the operation be done early the result is uniformly excellent. If it be deferred, in the hope that the child may "outgrow" its earaches, more or less impairment of hearing usually results, despite later treatment—even complete removal of the offending tissues, or the gradual atrophy of the hypertrophied structures in the course of time. To remove the tonsils alone is insufficient. The naso-pharynx and especially the fossa of Rosenmuller should be carefully cleaned of all excess of lymphoid structure under general anesthesia. I wish to state emphatically that the earlier this is done the better the result as to the preservation of normal hearing in adult life. I believe a large part of the many cases of incurable deafness of one or both ears we meet with in adults is directly due to neglect of the diseased condition I have just described. It is not safe to let the child "outgrow" its earaches.

So much for the significance of recurrent earache and its preventive treatment. Now how shall the family physician manage a case of earache during the attack.

You will recall that the tympanum is a small and narrow recess deep in the temporal bone, within the base of the pars petrosa. Its walls are bony and unyielding save the outer, which is largely membranous. At its lower anterior angle the Eustachian tube, closed when at rest or if inflamed, leads inward and a little downward to the naso-pharynx. Above and behind, it communicates through the antrum with the honeycomb of mastoid cells. Its roof, thin as a finger-nail and not infrequently dehiscent, alone separates it from the cranial cavity. You can readily understand how retention in this inelastic space of inflammatory products quickly causes exquisite pain, and how easily these same products, if the process continue unrelieved, may find their way into the mastoid cells or into the cranium itself—catastrophies to be prevented if possible.

Rich in nervous associations and blood-supply this region is readily influenced through the general circulation and nervous system, if inflammatory changes be not too advanced. The first indication, therefore, in the early stage of an earache, is to procure quiet, physical and mental. Place the patient at once in bed and keep him there, if a child, forty-eight hours at least. Many relapses occur from allowing a restless child to play about the house the next day after an earache. Let a hot footbath be given under the bedclothes for its relaxing and revulsive effect.

If a child from three to eight years of age, give morphia sulphate, gr. 1-16 to 1-10, by the mouth. Let the dose be large enough to be effective and given hypodermatically in older patients. The result sought is not only relief of pain, but to effect the circulation locally and in general. The opiate may be repeated if the pain recur or be not controlled, but it should not be continued the second day. There are then surgical means to be used to relieve pain, and at the same time to relieve tension in the tympanum and lessen the danger of mastoid or brain complications. Prolonged use of opium may mask serious symptoms until fatal mischief be done.

If the bowels are loaded or the tongue indicates it, give calomel and a saline in robust cases. Let a saline or other laxative, be given at least the next day after the morphia has been used. For two days let the patient have only soft food, milk toast, bread and milk, eggs, and the like, and light diet. The act of chewing tends to congest the ear and throat, and if solid food be masticated it may induce a return of pain. See that no erupting, or decayed, or ulcerated teeth are exciting inflammation in the ear. If present give them prompt attention. Let the room be kept at about 68 degrees and the air sweet and somewhat humid. This last is important where a furnace or natural gas supplies the heated air.

The local treatment of the early stage of acute catarrhal otitis is simple but important It consists of measures to apply warmth to the parts and to prevent sudden alternations of temperature. I prefer, as a rule, dry hot applications to moist ones. Let a large pad of wool or absorbent cotten be heated quite hot and applied over the entire affected side of the head. It can be held in place by a kerchief. In young children the pad can be stitched inside a little cap and effectively held in place. This should be worn during the time the patient is confined to bed.

Occasionally, if the pain continue despite the treatment already suggested, combined heat and moisture give relief. This is best applied by a very gentle stream from a fountain syringe. A quart at least of very hot water should pass steadily and slowly into the auditory canal to the fundus and then escape into a receptacle held below the ear. Some care is needed to apply this treatment properly. If it relieves pain it may be repeated as often as indicated.

Avoid the use of all applications about the auricle or within the auditory canal that may ferment or become rancid, such as sweet oil, oil and laudanum, and poultices of any kind. Such applications enormously increase the probability of serious infection of the tympanum from the auditory canal if the drum membrane rupture spontaneously or be incised to evacuate retained secretion.

Instillations of laudanum, morphine solutious, or cocaine solutions are of doubtful utility and they may be positively injurious in case of rupture of the drum-head. Their absorption is more than doubtful and their virtue consists largely in the warmth they convey.

There is frequently considerable naso-pharyngeal catarrh in these cases. Avoid the use of cleansing sprays or douches the first day or two of the attack. The congestion of the ear is very easily increased by them. A half of one per cent. solution of cocaine muriate in distilled water may be used if there be, as there often is, much nasal stenosis. This may be followed to advantage by a two per cent. solution of menthol in liquid alboline, sprayed slightly into nose and throat. Warn the patient against blowing the nose violently. Carelessness in this regard may cause a recurrence of the pain. Do not inflate the tympanum at this time by any method. That should be done only under expert advice.

If pain continues unabated or increased on the second day, do not trust longer to general medication or inexact applications, but call in an expert. The ear should then have a thorough skillful examination. Evacuation of the tympanic cavity by free incision of the drum membrane may be needed; and if promptly done, may prevent a prolonged purulent inflammation, with all the dangers it entails to hearing and even life itself. Do not trust to spontaneous rupture, or follow expectant treatment. It would be criminal negligence.

In summary, I beg to call your attention to the following points:

1. Earache, however slight, may signify disease that, neglected, may terminate in loss of hearing, even of life itself.

2. Recurring earache in children almost always is associated with lymphoid hypertrophy of the pharynx, depends on it, and permanent impairment of the function of the ear is prevented only by early surgical treatment of the "adenoids."

3. Acute inflammation of the middle ear may be frequently aborted if proper treatment—mostly of a general sedative character—be administered early in the attack and with precision.

4. If relief be not obtained by the second day an expert examination of the ear should be made and proper surgical treatment applied to relieve intratympanic pressure and possible involvement of the mastoid cells or intracranial structures. Failure at this stage to obtain as exact knowledge as possible of the condition of the middle ear is criminal negligence.

*305 Delaware Avenue.*

